# Exosomal circRNAs as promising liquid biopsy biomarkers for glioma

**DOI:** 10.3389/fimmu.2023.1039084

**Published:** 2023-04-14

**Authors:** Xiaoke Wu, Mengmeng Shi, Yajun Lian, Haifeng Zhang

**Affiliations:** ^1^Department of Neurology, The First Affiliated Hospital of Zhengzhou University, Zhengzhou, Henan, China; ^2^Department of Ophthalmology, The First Affiliated Hospital of Zhengzhou University, Zhengzhou, Henan, China

**Keywords:** liquid biopsy, exosomal circRNAs, glioma, biomarkers, exosome

## Abstract

Liquid biopsy strategies enable the noninvasive detection of changes in the levels of circulating biomarkers in body fluid samples, providing an opportunity to diagnose, dynamically monitor, and treat a range of diseases, including cancers. Glioma is among the most common forms of intracranial malignancy, and affected patients exhibit poor prognostic outcomes. As such, diagnosing and treating this disease in its early stages is critical for optimal patient outcomes. Exosomal circular RNAs (circRNAs) are involved in both the onset and progression of glioma. Both the roles of exosomes and methods for their detection have received much attention in recent years and the detection of exosomal circRNAs by liquid biopsy has significant potential for monitoring dynamic changes in glioma. The present review provides an overview of the circulating liquid biopsy biomarkers associated with this cancer type and the potential application of exosomal circRNAs as tools to guide the diagnosis, treatment, and prognostic evaluation of glioma patients during disease progression.

## Introduction

Tumors are formed by proliferating malignant cells caused by a range of oncogenic processes, including the accumulation of mutations and the abnormal proliferation of stem cells or specific tissues (1, 2). Changes in the expression of endogenous molecules or the production of entirely new molecules can be used as biomarkers of tumorigenesis (3). While histopathological biopsy remains the gold standard for the diagnosis of most tumor types, the inherent invasiveness of this approach limits its utility (4). While several tumor biomarkers are routinely analyzed in biofluid samples, including alpha-fetoprotein (AFP), carcinoembryonic antigen (CEA), carcinoembryonic antigen 125 (CA125), carcinoembryonic antigen 153 (CA153), glycoconjugate antigen 199 (CA199), and prostate-specific antigen (PSA), they are all subject to somewhat limited sensitivity and specificity such that their utility for early-stage tumor detection is limited (5–7). For example, while AFP is often upregulated in cases of hepatocellular carcinoma (HCC), this is not universally true (8, 9). Moreover, while CEA levels can be elevated in breast, pancreatic, or colorectal cancer, they may also be upregulated in nonmalignant diseases, including uremia, pulmonary fibrosis, and Alzheimer’s disease (10, 11). Increasingly advanced molecular detection techniques have led to the emergence of circulating tumor cells (CTCs), circulating tumor DNA (ctDNA), circulating tumor RNA (ctRNA), exosomes, and exosomal contents as promising tumor-related biomarkers that can be readily detected through the noninvasive sampling of biofluids – a process known as liquid biopsy. These liquid biopsy strategies have the potential to be more sensitive and specific than traditional oncogenic biomarkers, offering insight into tumor development, diagnosis, and prognostic evaluation (12–14).

**Figure 1:**
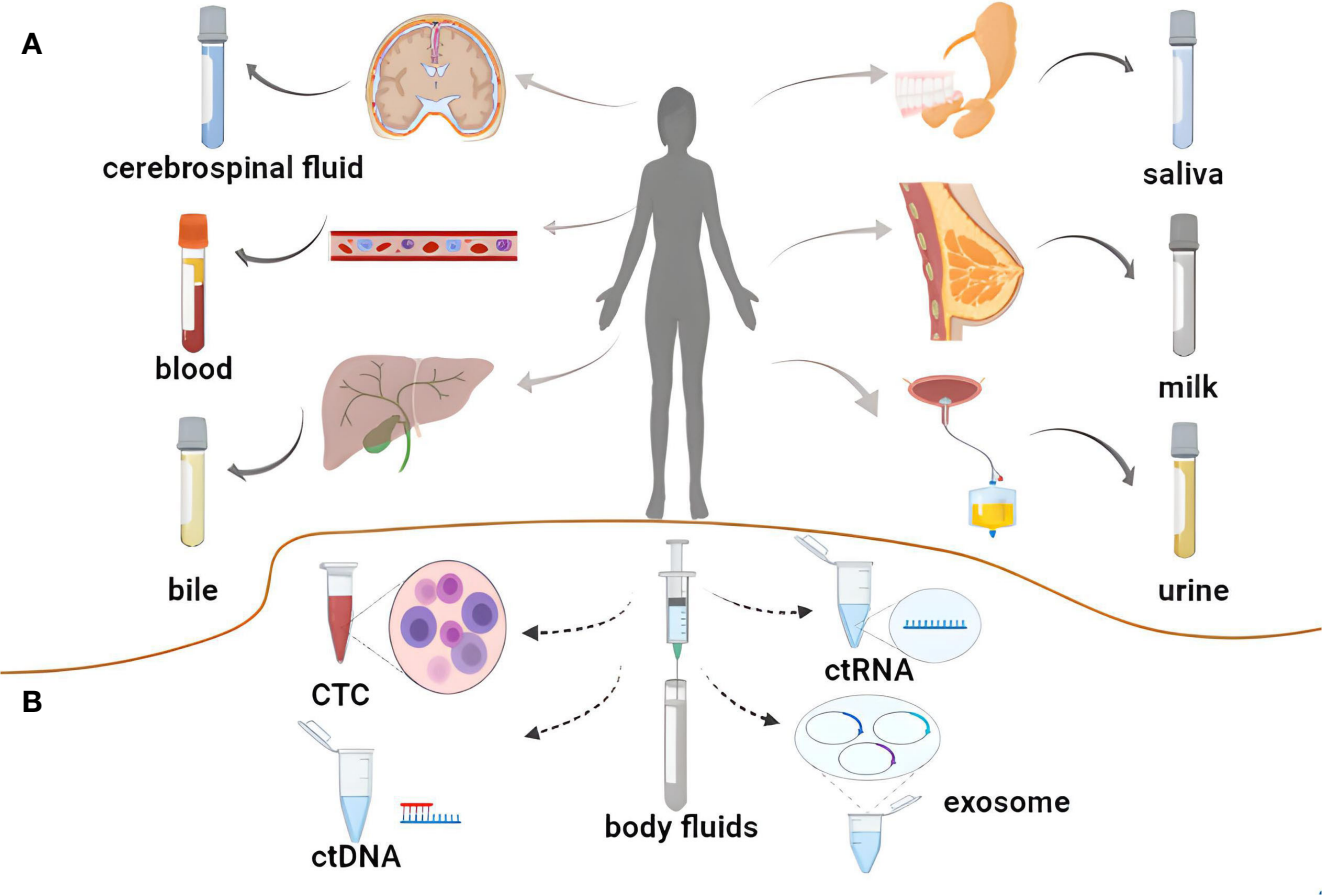
Liquid biopsy of tumors. **(A)** Body fluids used for liquid biopsy. **(B)** Novel biomarkers for liquid biopsies.

Liquid biopsy approaches enable the noninvasive assessment of tumor biomarkers in a range of biofluids including blood, saliva, cerebrospinal fluid (CSF), urine, bile, or breast milk (15–17). Exosomes are small (diameters: 40-150 nm) lipid bilayer-enclosed vesicles secreted by most cell types that contain a range of cargo molecules including lipids, proteins, specific cytokines, microRNAs (miRNAs), long non-coding RNAs (lncRNAs), and circRNAs (18, 19). Several studies have demonstrated that circRNAs can serve as important regulators of a diverse range of tumor-related processes such as proliferation, invasion, metastasis, stemness, and resistance to radiotherapy in part owing to their unique closed-loop structure (20). Exosomes can readily transport these stable circRNA molecules through biofluids including blood and urine, delivering them to recipient cells in which they can exert specific biological functions. As such, exosomal circRNAs have emerged as promising biomarkers and targets for therapeutic intervention for a range of tumor-related liquid biopsy assays (21, 22) (**Figure 1**).

Tumors of the central nervous system (CNS) are relatively rare but are associated with very high mortality rates (23). Gliomas comprise 81% of all CNS tumor cases, and are the second deadliest form of CNS disease (24, 25). Glioma cases are highly heterogeneous and the selection of appropriate treatment is highly dependent on pathological biopsy results, with most patients facing a poor prognosis (26). High levels of circRNAs have been detected in brain tissue where they have been found to influence tumorigenesis through the regulation of processes including inflammation, neuronal apoptosis, and angiogenesis (27, 28). By transmitting biological information between cells through exosome-mediated transmission, these circRNAs can profoundly shape glioma onset and progression (29). Assessing the onset, progression, therapeutic responsiveness, and prognosis of glioma cases is critical to guiding clinical care, and liquid biopsy analyses of exosomal circRNAs represent a promising approach to this form of disease monitoring (29, 30).

The present review summarizes the clinical application of exosomal circRNAs in the context of the early diagnosis, treatment, and prognostic evaluation of glioma patients through liquid biopsy-based approaches, with a particular focus on the relevance of exosomal circRNAs to the dynamic monitoring of disease progression with the goal of providing a robust foundation for future efforts to improve patient prognostic outcomes and to expand the clinical potential of liquid biopsy.

## Clinical applications of liquid biopsy strategies

The increased understanding of disease mechanisms together with technological development have led to significant advances in the diagnosis and treatment of a variety of diseases. Techniques such as blood or CSF analysis, tissue biopsy, MRI, and other imaging techniques have been widely used in clinical work. Analysis of blood and CSF are most commonly used; however, the tests tend to have poor specificity and often need to be combined with other tests for comprehensive judgment. Although more intuitive understanding of some diseases can be achieved through MRI and other optical techniques, they can only reflect the disease state at a specific stage. Tissue biopsy is the gold standard for disease diagnosis. However, although tissue biopsy is an accurate indicator of tumor diagnosis, it nevertheless has limitations. The use of exosome based-liquid biopsy is gradually achieving recognition. Liquid biopsies represent a convenient and minimally invasive alternative to traditional tissue biopsy techniques, enabling analyses of blood as well as other biofluids of interest such as the CSF, urine, bile, breast milk, or saliva. Notably, these approaches allow for the dynamic monitoring of tumors over the course of disease progression (15, 31). Beyond traditional tumor biomarkers, liquid biopsy approaches can also detect CTCs, ctDNA, ctRNA, exosomes, and exosomal contents (32).

## The application of liquid biopsy techniques in cancer

The production of CTCs, ctDNA, ctRNA, exosomes, and exosomal contents is highly heterogeneous over the course of tumor development and progression. Accordingly, the most appropriate methods for detecting these tumor-related biomarkers and their clinical significance when detected can vary substantially over the course of disease.

## CTCs in liquid biopsy

Initially identified in blood samples as early as 1860 (33), CTCs are small nucleated cells > 4 μm in size or clusters of 2-50 nucleated cells that express the epithelial protein EpCAM as well as the cytokeratins 8, 18, and/or 19. These CTCs also do not express cluster of differentiation 45 (CD45), which is an antigen specifically expressed by leukocytes, and there may be as many as several hundred CTCs per milliliter of blood in some cancer patients (34, 35). CTC is a cellular derivative of primary tumor tissue, and cancer cells enter body fluids from primary or metastatic solid tumors through the associated vascular or lymphatic system during the process of tumor metastasis (36, 37). Actual CTC counts in most body fluids are low, and the CellSearch assay is often used to detect these rare cells in diseases including breast, lung, liver, and neuroendocrine cancers (35, 38–41). In HCC, high levels of peripheral blood CTCs prior to treatment are closely related to the risk of microvascular invasion (MVI), and these postoperative CTC levels should be reduced. If they remain high (≥ 5), this can be indicative of a higher risk of early HCC recurrence (42). In patients with chronic obstructive pulmonary disease (COPD) at an elevated risk of lung cancer, CTCs can be detected through epithelial tumor cell size separation techniques up to 1-4 years before computed tomography (CT) scans can detect these tumors (43). As such, CTCs are highly sensitive tumor biomarkers.

## CtDNA detection in liquid biopsy samples

First described in 1940, ctDNA is DNA derived from tumor cells that circulates systemically, comprising a small fraction of the total cell-free DNA (cfDNA) (44). CtDNA is usually detected by next-generation sequencing or digital PCR (35) and is often utilized as a biomarker in liquid biopsy analyses aimed at diagnosing tumors, selecting appropriate therapeutic targets, detecting small residual tumors, or predicting the risk of disease recurrence (31, 45). The specific application of ctDNA has been used to guide the sensitive and specific detection of early-stage breast, colorectal, liver, lung, gastric, pancreatic, and ovarian cancer (46). Following surgical treatment, higher ctDNA levels in lung cancer patient plasma or breast cancer patient urine have been linked to a higher risk of recurrent disease, allowing for the detection of such risk at an earlier time point than that enabled by more conventional imaging strategies (47, 48).

## CtRNA detection in liquid biopsy samples

Any RNA derived from tumor cells, including messenger RNAs (mRNAs), miRNAs, circRNAs, and lncRNAs, is classified as a form of ctRNA when released into the systemic circulation. Reports of the detection of circulating mRNA in tumor patients were first published in the 1990s, and many more recent studies have similarly documented high levels of circulating miRNAs in tumor patient biofluids, with over 70 such miRNAs having been proposed as candidate HCC-related biomarkers (49). Higher levels of miR-10b and miR-21 in the CSF of glioblastoma patients with brain metastases have been described (50). LncRNAs are noncoding RNAs > 200 bp long that can be dysregulated in a manner conducive to tumor onset, progression, and metastasis. Accordingly, clinical efforts have recently focused on the successful diagnostic application of circulating lncRNAs in cancer (51). For example, higher plasma levels of the lncRNA CASC11 in early bladder cancer patients have been reported and found to coincide with plasma miR-150 downregulation (52). Additionally, circRNAs are highly stable single-stranded closed-loop RNAs that are highly abundant and stable in tissues and biofluids where they influence the progression of various diseases such as diabetes, neurological diseases, and cancer (53). Elevated serum circRNA404686 expression can offer value as a diagnostic biomarker of papillary thyroid microcarcinoma (PTMC) in women (54).

## Liquid biopsy analyses of exosomes

Exosomes are small lipid bilayer-enclosed vesicles derived from the endocytic pathway (55). They are present at high levels in most biofluids wherein they can transmit cargoes including proteins, circRNAs, miRNAs, and lncRNAs between cells (56). These exosomes can shape tumor progression through the promotion of angiogenic activity, the suppression of antitumor immunity, and the acceleration of metastatic tumor growth (57, 58). Studies have shown that the components of exosomes largely depend on their initiating parent cells (IPCs), carrying or mimicking the information of the parent cells. Thus, exosomes may represent useful diagnostic tools for cancers. In addition, non-invasive liquid biopsy for the detection of specific exosomes may be useful for both diagnosis and prognosis prediction (59, 60). Elevated exosomal levels are often detectable in liquid biopsy samples from cancer patients, including patients with breast or pancreatic cancer (61, 62). Ultracentrifugation, ultrafiltration, or related techniques can be used for the specific isolation of exosomes and other extracellular vesicles from biofluids of interest, thereby permitting the further interrogation of their cargo content (63, 64).

Exosomes are a type of extracellular vesicles (EVs) associated with both normal and diseased cells and have multiple biological functions. Exosome release is significantly increased in various diseases, including cancer. Various drugs that inhibit the release or uptake of pro-oncogenic exosomes in the tumor microenvironment (TME) have been proposed as novel cancer therapeutics (65, 66). The identification of different types of EVs can play an important role in the liquid biopsy analysis of malignant cancer. Hypoxic GM-derived (Glioblastoma cell, GM) exosomes have been found to contain significantly elevated levels of the monocarboxylate transporter 1 (MCT1) and its accessory protein differentiation cluster 147 (CD147) that promote the growth, metastasis, and invasion of GMs, as well as angiogenesis, in both *in vitro* and *in vivo* experimental models (67, 68). The levels of CD44 and CD133 were shown to be elevated in exosomes in anoxic environments, promoting glioma migration and lumen formation by endothelial cells.

Western blotting (WB), nanoparticle tracking analysis (NTA), dynamic light scattering analysis (DLSA), zeta potential analysis (ZPA), tunable resistive pulse sensing (TRPS), size exclusion chromatography (SEC), and other techniques can be used for the detection and characterization of EVs (69, 70). NTA and atomic force microscopy (AFM) can distinguish the size and concentration of exosomes and multivesicular vesicles (MVs, another type of extracellular vesicle). In addition, a localized surface plasmon resonance (LSPR) biosensor with self-assembly gold nanoislands (SAM-AuNIs) can be used to detect and distinguish EVs from MVs isolated from A-549 cells (67). Another LSPR method utilizes the self-assembly of a silver nanoparticle-decorated gold nano-island (Ag@AuNI) sensor chip to provide site-specific conjunction of biotinylated antibodies for the detection of exosomal surface biomarkers, and has high sensitivity and selectivity in the label-free sensing of exosomal biomarkers (71). Because exosomes originate from IPCs, it is particularly important to specifically identify their tissue of origin. Both LSPR and AFM have high sensitivity and specificity, and can detect and quantify raised levels of CD44 and CD133 in immunocaptured glioma-derived exosomes in the blood and CSF of animal models (72). In addition, biotinylated antibody-functionalized titanium nitride (BAF-TiN) and titanium dioxide (TiO2)-related LSPR can quantitatively detect exosomes isolated from human glioma cells, recognizing the exosomal marker membrane protein CD63, and BAF-TiN can also recognize glioma-specific variants of epidermal growth factor receptor variant-III (68). BIGH3 in GM-derived exosomes dynamically reflect the efficacy of TMZ (temozolomide) treatment of glioma, while TiO2-related LSPR can track the level of BIGH3 to monitor TMZ’s anti-cancer effect and prognosis after treatment (73).

The surface zeta potential (ZP) of exosomes sustains their stability, allowing the transfer of information between cells. Exosomes are released into the TME, leading to protein-protein and protein-lipid interactions and reducing the expenditure of energy. ATP produced by mitochondria and lactate accumulation may facilitate the entry of exosomes into cancer cells to promote metastasis and contribute to the targeting of cancer cells to the TME (74). Exosomes target the delivery of their cargoes by modifying various surface ligands, such as proteins, peptides, or aptamers. Therapeutic cargoes such as proteins, drugs, or small nucleic acids such as miRNA can be loaded in two different ways, both *in vivo* and *in vitro*. The source of the exosomes can affect the drug-loading capacity (75). Exosomes can cross the blood-brain barrier (BBB) to deliver drugs used for treating glioma. A microfluidic device (Exo-Load) can be used to improve the loading efficiency of doxorubicin (DOX) and paclitaxel (PTX) into glioma cell-derived exosomes, to improve the drug delivery efficiency (76).

## Liquid biopsy of exosomal circRNAs

High levels of circRNAs in serum exosomes were first reported in RNA-Seq analyses conducted in 2015, and they have since emerged as promising tumor-related biomarkers with the potential to guide diagnostic or related efforts (77). Mechanistically, circRNAs can exert diverse biological functions through interactions with particular RNA-binding proteins (RBPs) in a manner that enables the additional post-transcriptional or translational regulation of specific targets (78–80). In addition, circRNAs can function as molecular sponges capable of sequestering complementary miRNAs and preventing them from pairing with the 3’-UTR regions of their target mRNAs to inhibit translation, thus resulting in the indirect circRNA-mediated upregulation of these mRNA targets (81, 82). There is also evidence that some circRNAs can serve as templates encoding specific peptides related to tumorigenesis or disease progression (83). Tumor-related circRNA dysregulation has been confirmed through both animal studies and analyses of the serum exosomal circRNA profiles of healthy individuals and cancer patients (77). Exosomes are capable of delivering circRNAs to recipient cells, thus potentially driving tumor proliferation, therapeutic resistance, or metastatic progression (22). As such, liquid biopsy-based studies of exosomal circRNAs offer great potential for the dynamic monitoring of specific tumor-related biomarkers in glioma patients and individuals with related diseases.

## Liquid biopsy analyses of exosomal circRNAs in glioma

### Glioma

**Figure 2:**
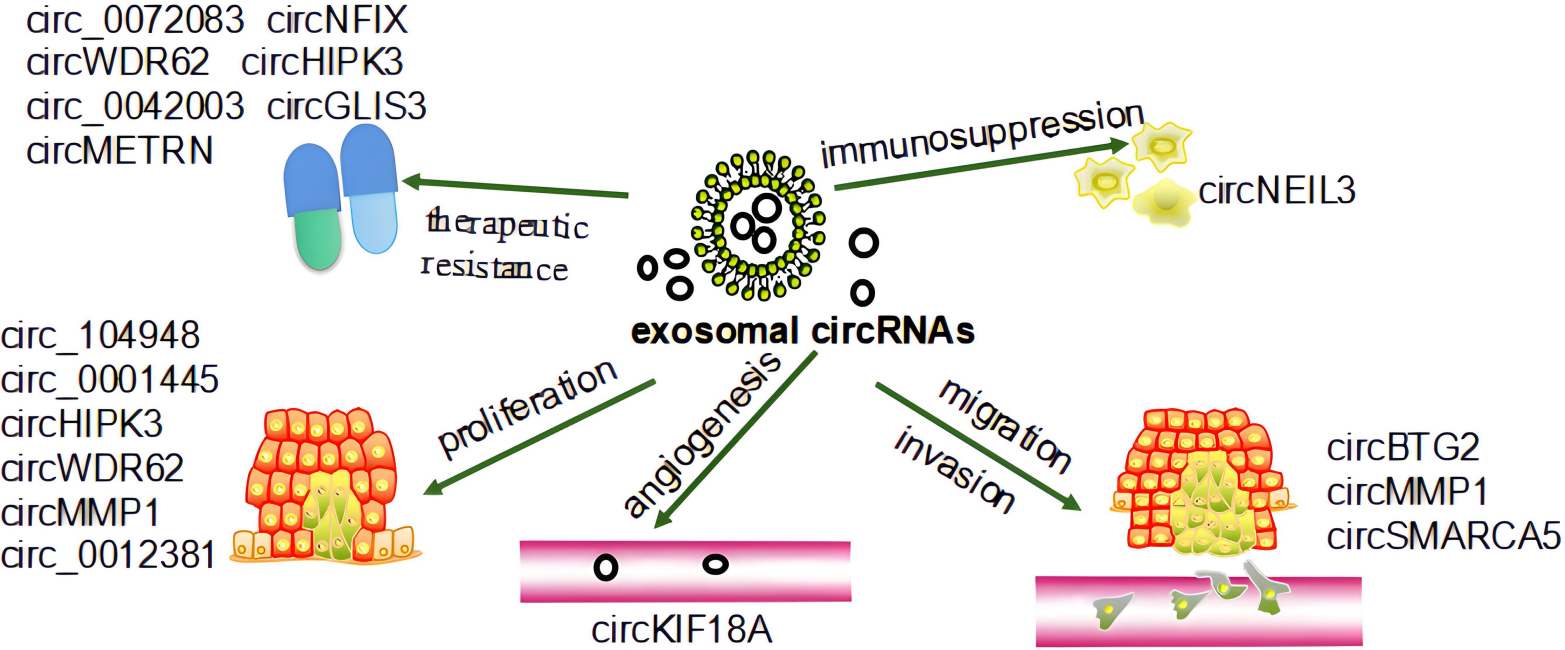
The role of exosomal circRNAs in the development of glioma. Exosomal circRNAs affect the progression of glioma by regulating its proliferation, invasion, migration, angiogenesis, and therapeutic resistance (30, 96–109).

Glioma is the most common CNS tumor and is characterized by rapid progression, aggressive growth, substantial heterogeneity, resistance to treatment, and poor prognostic outcomes (24, 84–86). The initial glioma diagnosis is generally guided by magnetic resonance imaging (MRI), However, accurate MRI-mediated tumor grading and analyses of tumor boundaries can be hampered by limitations associated with BBB permeability and the dilution of the contrast agent (87). The gold-standard approach to glioma diagnosis necessitates pathological biopsy, but these biopsy results have the potential to be inconclusive and may be contraindicated in some patients (88, 89). Many different processes drive glioma progression, and high levels of circRNA expression have been observed in brain tissue where they can act by sequestering specific miRNAs and thereby influencing tumor proliferative activity, invasion, and therapeutic sensitivity (90, 91). Significantly increased circPTN expression has been reported in glioma tissue and cell lines compared to healthy astrocytes, and the ability of circPTN to promote the proliferation of glioma cells by sequestering miR-145-5p and miR-330-5p has been demonstrated through both RNA immunoprecipitation and dual luciferase assays (92). The Notch signaling pathway is a key regulator of oncogenic pathways including cellular differentiation, apoptosis, and proliferation (93). Notably, the significant upregulation of circNFIX has been observed in glioma cells wherein it can regulate Notch1 by sequestering miR-34a-5p, thus influencing Notch signaling activity (94). As circRNAs are stably present in exosomes which are capable of readily crossing the BBB, they represent important intercellular communication targets that can serve as effective diagnostic, prognostic, or therapeutic biomarkers for the liquid biopsy-based evaluation of glioma patients (95). Exosomal circRNAs are present in the TME where they may play a vital role in the promotion of tumor progression. Exosomal circRNAs also play an important role in the development of glioma, including proliferation, immunosuppression, angiogenesis, invasion, migration, and therapeutic resistance. Recent studies have shown that exosome-associated circRNAs such as circ_104948, circ_0001445, circHIPK3, circWDR62, circMMP1, circ_0012381 play roles in the glioma proliferation process through miRNAs (30, 96–100). CircNEIL3 promotes glioma progression and exosome-mediated macrophage immunosuppressive polarization via stabilizing IGF2BP3 (101). Exosomal circKIF18A promotes angiogenesis by targeting FOXC2 in glioblastoma (GBM) (102). Macrophage-derived exosomes circBTG2 overexpression inhibited proliferation and invasion of glioma cells, circMMP1, and circSMARCA5 (circ_0001445) also play roles in the process of invasion and migration (30, 98, 103, 104). Low-dose radiation-induced exosomal circ-METRN plays an oncogenic role in glioblastoma progression and radioresistance through miR-4709-3p/GRB14 pathway; circ_0072083, circWDR62, circ_0042003, circNFIX, circHIPK3, circGLIS3 can resist TMZ in the treatment of glioma (96, 97, 105–109). **Figure 2** shows the roles by which exosomal circRNAs may be involved in the development of glioma.

### Exosomal circRNAs as diagnostic biomarkers for glioma patient liquid biopsy analyses

GBM is the most aggressive glioma subtype and accounts for roughly half of all cases. GBM patients exhibit lower levels of exosomal circSMARCA5 and circHIPK3 in their plasma samples, and researchers have reported improvements in GBM diagnostic accuracy when assessing these two exosomal circRNA biomarkers in combination with three traditional prognostic and diagnostic biomarkers associated with this tumor type including the preoperative neutrophil-to-lymphocyte ratio (NLR), platelet-to-lymphocyte ratio (PLR), and lymphocyte-to-monocyte ratio (LMR) (29, 110). GBM patients also reportedly exhibit significantly elevated plasma exosomal circ_0055202, circ_0074920, and circ_0043722 levels, and inhibiting the expression of these three circRNAs in the U87 cell line significantly impaired their proliferative activity, suggesting that this may be a viable biomarker for the detection of GBM (111). High levels of serum exosomal circMMP1 (circ_0024108) have been reported in glioma where it can promote glioma cell proliferative and migratory activity while inhibiting apoptotic cell death (30). High levels of circFBXW7 expression are evident in healthy brain tissue where it encodes the FBXW7-185aa protein. However, the upregulation of FBXW7-185aa in tumor cells can suppress proliferation, and as a result, GBM tissues have been shown to have lower levels of circFBXW7 and FBXW7-185aa expression relative to adjacent tissues. A positive correlation between circFBXW7 expression and overall survival has been reported in GBM patients, with corresponding circFBXW7 downregulation in glioma tissues (112, 113). In addition, the downregulation of CDR1as has been observed in glioma wherein it has been shown to bind the DNA-binding domain of the p53 tumor suppressor protein, thus inhibiting the ubiquitination and degradation of p53. Accordingly, inactivating CDR1as can promote gliomagenesis (114, 115).

### Exosomal circRNAs as therapeutic markers for glioma patient liquid biopsy analyses

CircMMP1 can promote the proliferation of tumor cells while inhibiting apoptosis, and high levels of exosomal circMMP1 are evident in the glioma where it promotes oncogenesis via the circMMP1/miR-433/HMGB3 axis, indicating that this pathway may be amenable to patient treatment (30). In glioma patients undergoing radiotherapy, exosomal circATP8B4 can sequester miR-766 and contribute to radioresistance (116). The expression of circHIPK3 is increased in glioma cells and tissues where it promotes miR-124 sequestration-mediated enhancement of CCND2 expression, ultimately driving increased proliferation and invasion. Exosomal circHIPK3 can promote the proliferation of tumor cells and resistance to TMZ treatment through the miR-421/ZIC5 pathway (97, 117). TMZ-resistant glioma patients also exhibit higher levels of exosomal circNFIX, which can promote oncogenic progression. The upregulation of circNFIX reduces glioma cell sensitivity to TMZ as a consequence of miR-132 downregulation (107). The upregulation of circ_0042003 in exosomes derived from the TMZ-resistant U251 cell line can be positively regulated by acetyl heparinase, which influences glioma cell TMZ resistance and can be knocked down to sensitize U251 cells to this drug (109). Higher levels of circ_0072083 in TMZ-resistant glioma tissues and cells can regulate ALKBH5 via miR-1252-5p, thereby influencing TMZ resistance. The Warburg effect can promote exosomal circ_0072083 release from drug-resistant cells, and this circRNA can, in turn, enhance the TMZ-resistant properties of sensitive cells (106).

### Exosomal circRNAs as a prognostic marker for liquid biopsy of glioma

**Figure 3:**
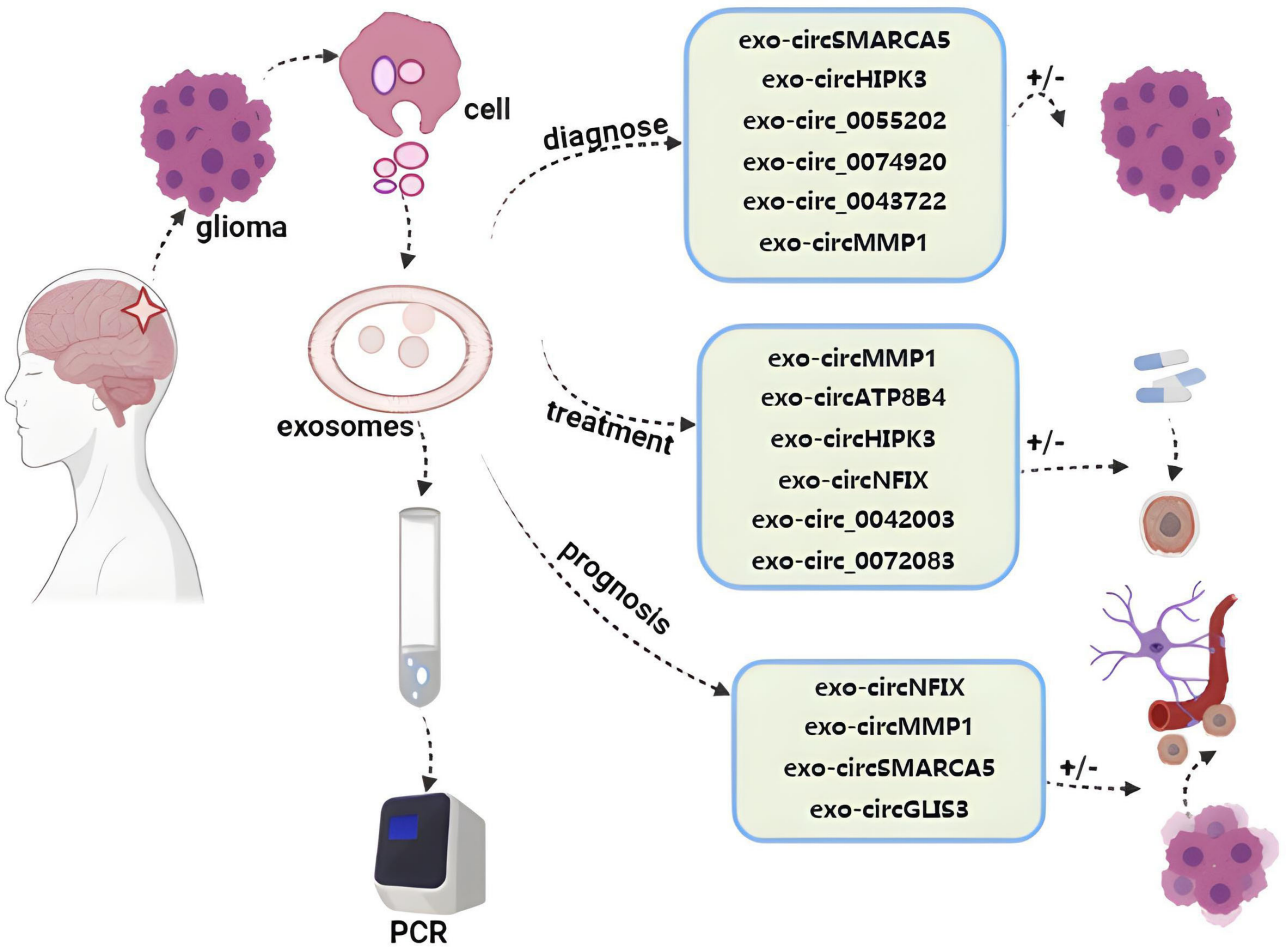
Roles of exosome circRNAs in liquid biopsy for glioma. By detecting exosomal circRNAs evaluating diagnosis, treatment, and prognosis of glioma (29, 30, 97, 98, 103, 107, 109, 113–115, 117, 119).

High serum exosomal circMMP1 (circ_0024108) levels have been potentially linked to poor glioma patient prognosis (30). While higher levels of exosomal circNFIX have been associated with resistance to TMZ treatment, suggesting that analyses of this circRNA can better guide the care and therapeutic monitoring of affected patients (107, 118). The positive cell migration regulator splicing factor 1 (SRSF1) is expressed at high levels in glioblastoma cells, and it exhibits multiple binding sites for circSMARCA5. Moreover, higher levels of splicing factor 3 (SRSF3) expression are observed in glioma and it has been speculated to function as a positive regulator of SRSF1-dependent glioma cell migratory activity. Lower levels of exosomal circSMARCA5 can inhibit the migration of GBM cells through the regulation of the SRSF1/SRSF3/PTB axis, indicating that it may function as a potent tumor suppressor in this cancer type through the inhibition of SRSF1 function (103). In addition, the upregulation of circGLIS3 is evident in high-grade glioma and exosomes can facilitate its secretion into the glioma microenvironment, facilitating tumor invasivity, angiogenic activity, and the phosphorylation of Ezrin (T567) phosphorylation. Elevated levels of p-Ezrin (T567) are, accordingly, associated with high-grade glioma and with a poor patient prognosis (119) (**Figure 3**).

## Conclusions and outlook

Given their noninvasive nature and amenability to convenient sample collection and analysis, liquid biopsy-based testing strategies have developed rapidly in recent years and are used in a range of advanced clinical tests assessing particular biomarkers relevant to specific disease states. Liquid biopsy strategies can be used for early cancer screening and diagnosis, while also enabling the prediction of therapeutic targets, therapeutic resistance, post-treatment follow-up outcomes, and disease recurrence. Many recent studies have explored the molecular basis for the pathogenesis of glioma, spurring the increased application of liquid biopsy-based analyses in this oncogenic context. Exosomal circRNAs have emerged as particularly promising biomarkers in the context of liquid biopsy analyses, offering higher levels of sensitivity and specificity compared with more traditional biomarkers, thereby providing clinicians with better guidance for the timely diagnosis, prognostic assessment, and monitoring of treatment resistance in individuals affected by glioma. At present, the methods for detecting exosomes are increasing, together with improved specificity and credibility, leading to potential for borad clinical application. Through the discussion of the formation, action mechanism, clinical application and detection means of exosomes, we have gained a deeper understanding of the role of exosomes in disease. CircRNAs in exosomes can play a role in both the physiological and pathological state of the body, especially in tumors. The deepening understanding of the roles of exosomal circRNAs in glioma has led to the proposal of novel clinical applications. Exosomal circRNA is a small molecule present in body fluids where it can be accurately detected by liquid biopsy technology, allowing its precise analysis.

Liquid biopsy strategies are still subject to a range of challenges and necessitate further validation before they can enter into routine clinical use. For one, it is important that appropriate biofluids be selected when conducting these analyses, and whether a given biomarker is the most appropriate target in that biofluid sample also warrants consideration. One must also consider whether the source of a given biomarker detected in a particular biofluid is unique to primary or metastatic tumor-derived sources or whether it may also arise from other tissues or cell populations in the body. Given the relatively low levels of particular molecular biomarkers in samples of different body fluids, this can place substantial demands on the volume of available body fluids, and highly sensitive assay instruments must be capable of detecting even small changes in these biomarker concentrations. As they are a relatively new type of tumor marker, there also remain several barriers to the clinical application of exosomal circRNAs, and new detection instruments with higher levels of sensitivity will be essential to facilitate their routine detection. It is also important that the molecular basis of glioma pathogenesis be better understood, so that exosomal circRNAs can be more reliably applied to guide the diagnosis and treatment of patients suffering from this devastating form of cancer. We believe exosomal circRNAs can be used as liquid biopsies and noninvasive biomarkers for the early detection, diagnosis, and treatment of cancer and other diseases in the nearly future.

## Author contributions

YL and HZ provided direction and guidance throughout the preparation of this manuscript. XW wrote and edited the manuscript. MS reviewed and made significant revisions to the manuscript. All authors contributed to the article and approved the submitted version.
